# 

**Published:** 1919-09

**Authors:** 


### ANNOUNCEMENTS

### NATIONAL SOCIETIES

AT NEW ORLEANS, OCTOBER 17 to 24, 1919

17-18—International Society of Prosthetists.
18-20—National Association of Dental Examiners.
20-24—National Dental Association.
20-24—National Association of Dental Faculties.
20—Delta Sigma Delta.
20-24—Xi Psi Phi Fraternity.
20-24—Association of Military Dental Surgeons.
20-24—Psi Omega Fraternity. Grand Chapter meeting and National Alumni Chapter meeting.

### PRELIMINARY ANNOUNCEMENT OF THE 1919 PROGRAM OF THE NATIONAL DENTAL ASSOCIATION, NEW ORLEANS, LA.

*Program of the Section on Orthodontia and Periodontia, October* 20-24, 1919

*Tuesday Afternoon,* 2:00 *p. m.*—"An Analysis of the Various Principles of Orthodontic Treatment That Have Been Advocated During the Last Fifteen Years," by J. A. Cameron Hoggan, Richmond, Va. "Necessity for Oral Prophylaxis and Radiology in the Practice of Orthodontia," by F. M. Casto, Cleveland, Ohio. "Observations upon the More Recent Developments in Periodontology," by Arthur H. Merritt, New York, N. Y. "Why the Field of Prophylaxis is Marked with a Lack of Enthusiasm," by Dorothea A. Howes, Washington, D. C.

*Wednesday Morning,* 9:00 *a. m.*—"Plurality of Etiology of Periodontoclasia," by John O. McCall, Buffalo, N. Y. "What to Extract, and What not to Extract, with Referenec to Infections Involving the Periodontal Membrane," by Louis D. Corriell, Baltimore, Md. "An Analysis of Case Characteristics with Reference to the Selection of the Most Efficient Form of Appliance for Treatment," by A. H. Ketcham, Denver, Colo. "The Problem of Retention," by C. A. Hawley, Washington, D. C.

*Program of the Section on Operative Dentistry, Materia Medica and Therapeutics, October* 20-24, 1919

*First Session.*—"The Prevention of Chronic Mouth Infection," (illustrated with stereopticon slides), by Arthur D. Black, Chicago, Ill.; discussed by H. E. Friesell, Pittsburgh, Pa.; A. H. Hipple, Omaha, Neb. "X-Ray in Dental Practice," by C. Edmund Kells, New Orleans, La.; discussed by H. B. Tileston, Louisville, Ky.; Howard R. Raper, Albuquerque, N. M.

*Second Session.*—"Some Recommendations for the Sterilization and Filling of Infected Roots," by Weston A. Price, Cleveland, O.; discussed by Clarence J. Grieves, Baltimore, Md.; Percy R. Howe, Boston, Mass. "The Gold Inlay," by R. H. Volland, Iowa City, Iowa; discussed by Wallace Wood, New Orleans, La.; W. L. Fickes, Pittsburgh, Pa.

*Program of the Section on Histology, Physiology, Pathology, Bacteriology and Chemistry—Research, October* 20-24, 1919

*First Session.*—"How Mouth Infection Affects the Kidneys," by Thomas B. Hartzell, Minneapolis, Minn. "A Phase of Dental Caries," by Percy R. Howe, Boston, Mass. "A Biochemical Study of Bacterial Metabolism in Its Relation to the Denser Tooth Structures," by Samuel E. Pond, Cleveland, Ohio.

*Second Session.*—"A Dental Histo-Pathological Study," by Harold Box, Toronto, Canada. "Physiology and Pathology of Special Interest to Dentists," by J. J. Sarrazin, New Orleans, La. "Studies of the Variations in Susceptibility to the (so-called) Rheumatic Group Lesions and to the Influence of Oral Focal Infections,'' by Weston A. Price, Cleveland, Ohio.

*Program of the Section on Prosthetic Dentistry and Crown and Bridge Work, October* 20-24, 1919

FIRST SESSION—*Prosthodontia.*—''Scientific Interpretation of Muscular Control of Mandibular Movements,'' by George H. Wilson, Cleveland, Ohio. ''Surgical Interference for Preparation of Malformed Edentuous Mouths for Construction of Dentures,'' by James P. Ruyl, New York City.

*Crown and Bridge Technic.*—''Construction of Cast Clasps for Partial Dentures and General Consideration of Other Methods of Retention and Attachment of Vital Teeth for Removable Bridge Work,'' by Louis J. Weinstein, New York City. ''Porcelain Jacket Crown Technic,'' by A. L. Legro, Detroit, Mich.

SECOND SESSION—*Prosthodontia.*—''Selection of Artificial Teeth for Prosthetic Restorations,'' by P. C. Lowery, Detroit, Mich. ''Correction of Malocclusion in Artificial Dentures,'' by M. M. House, Indianapolis, Ind.

*Crown and Bridge Technic.*—''The Gold Shoulder Crown Technic,'' by Wm. H. Elliott, Detroit, Mich. ''Attachments for Vital Teeth in Fixed Bridge Work,'' by Forrest H. Orton, St. Paul, Minn. ''Mandibular Movements and the Forms of Artificial Bicuspids and Molars Necessitated Thereby,'' by Prof. Alfred Gysi, Zurich, Switzerland. ''Mandibular Control,'' by J. Leon Williams, New York City.

*Program of the Section on Oral Surgery, Exodontia and Anesthesia, October* 20-24, 1919

*First Session.*—Symposium: ''Apicoectomy.'' ''Its Indications and Contraindications and Root Canal Technic,'' (illustrated with stereopticon slides), by Thomas B. Hartzell, Minneapolis, Minn. ''Surgical Technic of Apicoectomy'' (illustrated with stereopticon sides), by Chalmers J. Lyons, Ann Arbor, Mich; discussed by Thomas P. Hinman, Atlanta, Ga.; William L. Shearer, Omaha, Nebr.; Carl D. Lucas, Indianapolis, Ind.; Clarence J. Grieves, Baltimore, Md.; H. A. Maves, Minneapolis, Minn. ''Nitrous Oxid-Oxygen Anesthesia in Oral Surgery and Dentistry,'' by J. A. Heidbrink, Minneapolis, Minn.; discussed by Wm. H. Deford, Des Moines, Iowa; John W. Seybold, Denver, Colo.; Boyd S. Gardner, Rochester, Minn. ''Tic doloureaux —Etiology—Diagnosis—Treatment—Palliative — Blocking and Surgical,'' by Rudolph Matas (M.D.), New Orleans, La.; discussed by Herbert A. Potts, Chicago, Ill.

*Second Session.*—''Impacted Lower Third Molar'' (illustrated), by George B. Winter, St. Louis, Mo.; discussed by J. P. Henahan, Cleveland, Ohio; Harry W. Allen, Kansas City, Mo.; O. T. Dean, Seattle, Wash.; Roy S. Hopkinson, Milwaukee, Wis. Symposium: ''Block Anesthesia.'' ''Preparation of Solution,'' by E. A. Litchfield, Humboldt, Nebr. ''Pharmacology of Various Local Anesthetics,'' by Samuel L. Silverman, Atlanta, Ga. ''Indications and Contraindications,'' by Fred F. Molt, Chicago, Ill. ''Technic of Blocking'' (most important injections), by ————. ''Suggestive Therapy and Treatment of Abnormal Conditions During and Following Injections,'' by P. G. Puterbaugh, Chicago, Ill. ''Diseases of the Antrum'' (illustrated with stereopticon slides), by Charles H. Oakman, Detroit, Mich.; discussed by R. Boyd Bogle, Nashville, Tenn.; Truman W. Brophy, Chicago, Ill.

